# Experimental evidence that a combination of sGC stimulator BAY 60-4552 and PDE5 inhibitor vardenafil could salvage ED-patients with insufficient response to PDE5 inhibitors after cavernous nerve injury

**DOI:** 10.1186/1471-2210-11-S1-P59

**Published:** 2011-08-01

**Authors:** Alexandra Oudot, Delphine Behr-Roussel, Sarah Poirier, Jacques Bernabé, Peter Sandner, François Giuliano

**Affiliations:** 1 Pelvipharm, Orsay, France; 2 Bayer HealthCare AG, Global Drug Discovery, Wuppertal, Germany; 3 AP-HP, Neuro-Uro-Andrology, Dept. of Physical Medicine and Rehabilitation Raymond Poincaré Hospital, Garches, France; 4 EA 4501 Université Versailles Saint Quentin en Yvelines, Garches, France

## Background

Radical prostatectomy (RP) is frequently responsible for erectile dysfunction (ED). Post-RP patients often show insufficient response or treatment failures to PDE5 inhibitor therapy. This study was undertaken to evaluate the acute effects of the soluble guanylate cyclase (sGC) stimulator, BAY 60-4552 and vardenafil administered alone or in combination on erectile responses to electrical stimulation of the cavernous nerve (ES-CN) in rats with cavernous nerve (CN) crush injury-induced ED.

## Design and methods

Male Sprague-Dawley rats underwent laparotomy (sham, n=10) or bilateral CN crush injury (n=57). After 3 weeks of recovery, erectile function was evaluated in urethane-anesthetized rats following ES-CN at different frequencies. The acute effects of intravenous (iv) injection of vehicle, vardenafil 0.03 mg/kg, BAY 60-4552 0.03 mg/kg or 0.3 mg/kg, or a BAY 60-4552 0.03 mg/kg + vardenafil 0.03 mg/kg combination were evaluated in CN crushed rats.

## Results

Bilateral CN crush injury followed by a 3-week recovery period decreased erectile responses to ES-CN by about 50%. In CN crushed rats, both iv vardenafil 0.03 mg/kg and BAY 60-4552 at the tested dosings (0.03 or 0.3 mg/kg) increased erectile responses to ES-CN to the same extent: ΔICP/MAP at 10Hz ES-CN was 20.9± 1.3 % after iv vehicle, 25.3± 3.3 % (P<0.001) after iv vardenafil, and 26.3± 4.9 % and 26.6± 5.2 % after BAY 60-4552 0.03 mg/kg (P<0.01) and 0.3 mg/kg (P<0.001) respectively. The combined iv administration of vardenafil and BAY 60-4552 in CN crushed rats exerted additive effects and totally restored erectile responses to ES-CN equivalent to sham rats (ΔICP/MAP at 10Hz ES-CN : 34.0± 4.4 % after BAY 60-4552/vardenafil combination vs 39.2± 3.7 % in sham rats, ns).

## Conclusion

The present study supports the concept that the combined administration of a sGC stimulator, BAY 60-4552 and vardenafil provides additive beneficial effects. Thus this combination could become a novel treatment option in ED patients with cavernous nerve injury showing insufficient response or treatment failures to PDE5 inhibitors.

